# A novel heterogeneous biocatalyst based on graphene oxide for synthesis of pyran derivatives

**DOI:** 10.1038/s41598-024-57682-y

**Published:** 2024-03-23

**Authors:** Leila Amiri-Zirtol, Soghra Khabnadideh

**Affiliations:** Pharmaceutical Sciences Research Center, Shiraz University of Medical Sciences, Shiraz, Iran

**Keywords:** Medical research, Chemistry

## Abstract

Graphene oxide modified with tryptophan (GO-Trp) has been introduced as a new heterogeneous acid-base biocatalyst for synthesis of some pyran derivatives. GO was prepared according to the Hummer’s method and tryptophan as a low-cost green amino acid is covalently bonded to the surface of GO without any organic or toxic reagents in a green way. The new catalyst was characterized by different spectroscopic methods such as Fourier transform infrared, X-ray diffraction (XRD), etc. …. The results of XRD patterns showed an increase in the distance between the GO plates in the presence of the modifying agent which specifies the presence of amino acid between the GO layers. XPS analysis also confirmed successful modification through the presence of C–N bonds in the structure of the catalyst. In addition, improvements in thermal stability and changes in the morphology of the samples were observed using thermogravimetric analysis and Field emission scanning electron microscopy analysis respectively. Evaluation of the catalyst performance in the synthesis of some benzo[*b*]pyran and pyrano[3,2-*c*] chromene derivatives showed presentable results. Seven benzo[*b*]pyran (***4a–4g***) and five pyrano[3,2-*c*] chromene (***4h–4l***) derivatives were synthesized. GO-Trp as a safe, natural and efficient catalyst, could be reused up to 5 runs for synthesis of pyran derivatives without any significant decrease in its potency. High purity of the products and desirable yields are other points that make the present work more attractive.

## Introduction

In recent decades, using of organocatalysts has been announced as a successful strategy for the greener synthesis of organic compounds^1,2^. These catalysts are small molecules, inexpensive, available and efficient organic molecules with sufficient stability and economic efficiency. They are widely used in chemical reactions while there is no need to metal for their activation^1,3,4^. Recently, the design and performance of organic–inorganic nanocomposite materials have been extensively established, mainly focusing on the ability to control their nanoscale structure via innovative synthetic approaches^5^. Amino acids as biocatalysts have both carboxylic acid and amino functional groups which led to the use of these natural compounds as dual action catalysts. The ability to easily recover and reuse the catalysts in organic evolution is highly desired economically and environmentally^6–8^. For this purpose, the use of backup is widely interested in the preparation of these catalysts^9,10^. Of the numerous known supports to date, natural-based compounds have received the most attention due to their alignment with green chemistry. Among these, carbon-based materials are very prominent due to their diverse structures. GO is one of these compounds which have a two-dimensional structure and a layer consisting of carbon, oxygen and hydrogen atoms. Having a large surface area, multiple functional groups and low intrinsic mass are some of the reasons for using GO as a strong heterogeneous support^11–13^. The specific structure of GO causes it to interact with a wide range of organic molecules through covalent or non-covalent bonds including hydrogen bonding, accumulation, hydrophobic relationships, electrostatic and van der Waals forces^14,15^. There are some reports about the respective effect of graphene and GO on human health. These carbon nanomaterials have a certain effect on human health, plants and animals. As these nanomaterials have been widely studied and applied, their effect and the potential future impact on aquatic environment should not be ignored and the knowledge about their fundamental toxicity is needed^16^.

Tryptophan is an essential amino acid in the human body, containing NH_2_, COOH groups, and also an indole moiety in its structure. Its acidic part (–COO^−^) has pKa = 2.38 and its amine part (–NH^+^) has pKa = 9.39. Despite its interesting structure and catalytic potential, its use as a heterogeneous organocatalyst in the synthesis of organic molecules is less common than the other amino acids. Aghapoor et al.^17^ introduced tryptophan as a homogeneous catalyst for cyclo-condensation reactions. In 2019, Ghorbani et al.^18^ used tryptophan and palladium to design a new metal catalyst to form a carbon–carbon bond.

On the other hand, currently, multi-component reactions are widely used as a successive strategy to synthesize different organic compounds particularly in the case of heterocycles^19^. Multi-component reactions are a group of chemical reactions in which primary substances react together and create a single product. These reactions have benefits such as easy method, high atom economy, reduction of waste generation and complex purification procedures^20–28^. Oxygenated heterocyclic compounds such as pyrans and chromones have been synthesized by using this method as well. These compounds have various pharmacological properties such as anti-cancer, anti-inflammatory^29–33^, anti-coagulation^34^, anti-anaphylactic^35^ and anti-Alzheimer^36^. These are important structural units found widely in natural products^37^. So far, various catalysts^32–34,38–42^ have been introduced for the synthesis of these compounds. Maleki et al.^43^ introduced core–shell Fe_3_O_4_@SiO_2_-creatine as a heterogeneous catalyst for synthesis of pyran derivatives. But this catalyst was synthesized via several steps which show hard and expensive process for preparation of this catalyst. However, some of the reported methods have drawbacks such as long reaction time, expensive reagents and long process for the synthesis of the catalyst^44^.

Here we wish to introduce a novel heterogeneous biocatalyst using of tryptophan which is stabilized on the surface of GO sheets via a cost-effectiveness of method. Tryptophan was stabilized on the surface of GO by creating covalent bonds between amino acids and GO’s functional groups. This heterogeneous catalyst was synthesized in a simple and green way in an aqueous media at room temperature that are noteworthy points. Using an easy and clean method with inexpensive and available materials are new strategies that are introduced in this study. As far as we know, there are no reports of tryptophan stabilization on GO. The synthesized catalyst was identified and investigated by FT-IR, XRD, X-ray photoelectron microscopy (XPS), Energy-dispersive X-ray (EDX), Raman, TGA and FE-SEM analysis. All results confirmed the placement of tryptophan on GO sheets. After decoration of tryptophan on GO and binding it to nitrogen, the amount of free carboxylic acid groups is increased. The new catalyst was used for synthesis of pyran derivatives by multicomponent reactions. Some benzo[*b*]pyran (***4a–4g***) and pyrano[3,2-*c*] chromene (***4h–4l***) derivatives were synthesized, at room temperature and green environments with high efficiency and easy separation. Chemical structure of the pyran compounds were confirmed by m.p., TLC and for some of them by spectroscopic methods. Good efficiency and short reaction time in the synthesis of pyran derivatives confirmed the high acidity of the catalyst in comparison to the GO.

In this study, tryptophan, as an amino acid, is immobilized on the surface of GO for the first time in an environmentally benign manner. The new composite was used as a novel bioorgano-catalyst for the synthesis of pyran compounds.

## Results

In this research a new heterogeneous biocatalyst was introduced and identified. The catalyst capability for synthesis of some pyran derivatives under moderate conditions was evaluated as well. For synthesis of the catalyst, GO prepared according to the Hummers’ method. Then tryptophan was decorated on the surface of GO in water at room temperature without any toxic or hazardous materials. The prepared composite showed significant and acceptable catalytic power in the synthesis of benzo[*b*]pyran and pyrano[3,2–*c*] chromene derivatives under moderate conditions. Immobilization of the amino acid on the surface of GO according to the epoxy groups on the surface was done easily without any toxic activators such as thionyl chloride. Using of GO also has an important role in the proper decoration of tryptophan due to its large surface area. The binding of this amino acid via covalent bonds causes the stability of the catalyst for reuse in the next reactions. The positive effect of tryptophan on the catalytic activity of the catalyst is apparent by comparison of the reaction efficiency in the presence of GO with GO-Trp. As the acidity of GO is not sufficient to carry out the reaction, its binding to tryptophan contributed to having more acidic character which led to act as a more powerful catalyst.

### Catalyst identifications

#### FT-IR analysis

**Figure 1:**
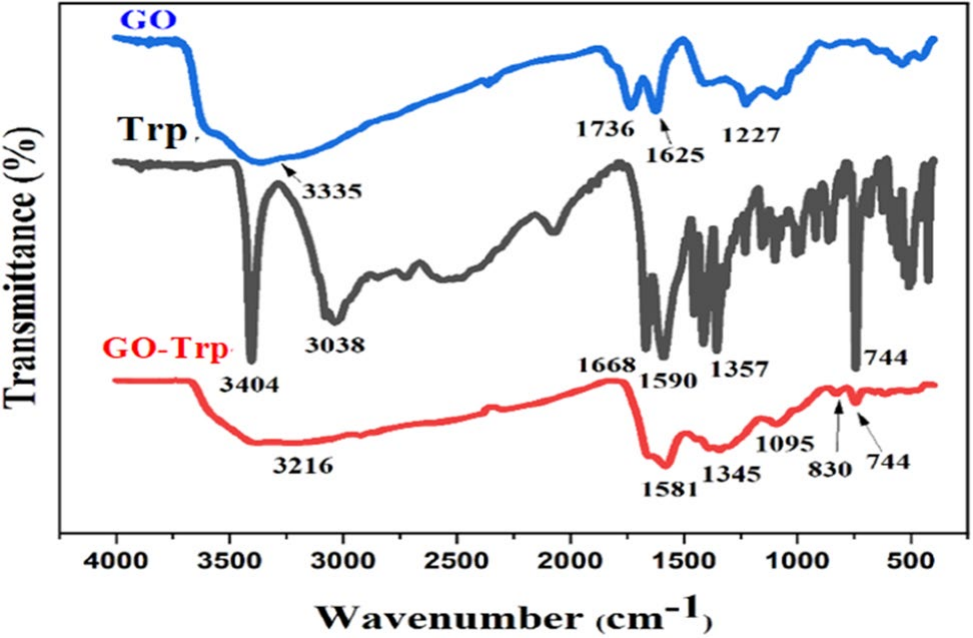
FTIR spectra of GO, Trp, and GO-Trp.

The FT-IR spectra of the GO, Trp, and GO-Trp were shown in Fig. 1. The FT-IR spectrum of GO showed certain groups with bands in certain areas. The GO absorption band in the region of 3337 cm^−1^ correspond to the stretching vibrations of the OH groups on the sheet and the carboxylic acid of the GO wall. Bands in the 1736 and 1625 cm^−1^ areas are related to the stretching vibrations of the C=O and C=C groups, and bands related to the C–O and alkoxide groups were observed in the range of 1227 cm^−1^^45^. FT-IR spectra of the amino acid showed two bands in 3404 and 3038 cm^−1^ belonging to the N–H of the indole ring and the stretching vibrations of the O–H carboxylic acid groups, respectively. The bands in the 300–2800 and 1668 areas are related to the C–H asymmetric stretching vibration and the NH_2_ stretching vibration, respectively. Absorptions in the range of 400–1300 cm^−1^ are related to the other functional groups such as CNH stretching and COO^−^ asymmetric and symmetric stretching. By placing the amino acid on the GO sheets, certain changes such as displacement, removal and formation of some new bands were clearly visible in the related spectrum. The absorption intensity of bands at 3216 cm^−1^ for O–H and N–H stretching, at 1736 cm^−1^ for C=O, and at 1227 cm^−1^ for C–O of epoxy were decreased, which indicated GO surface changes. At 1000 to 1561 cm^−1^, the tryptophan bands interfered with GO bands and changed the GO spectrum. The band appearing in the range 1615–1630 cm^−1^ is related to the stretching vibration of the C=C groups, and N–H, which overlaps with the band corresponding to the –COO^−^ groups. The band appearing in 1345 cm^−1^ belonged to the bending vibration of the CH_3_ groups. The resulting spectrum showed that the organic compound is located on the surface of GO.

#### Morphological studies

**Figure 2:**
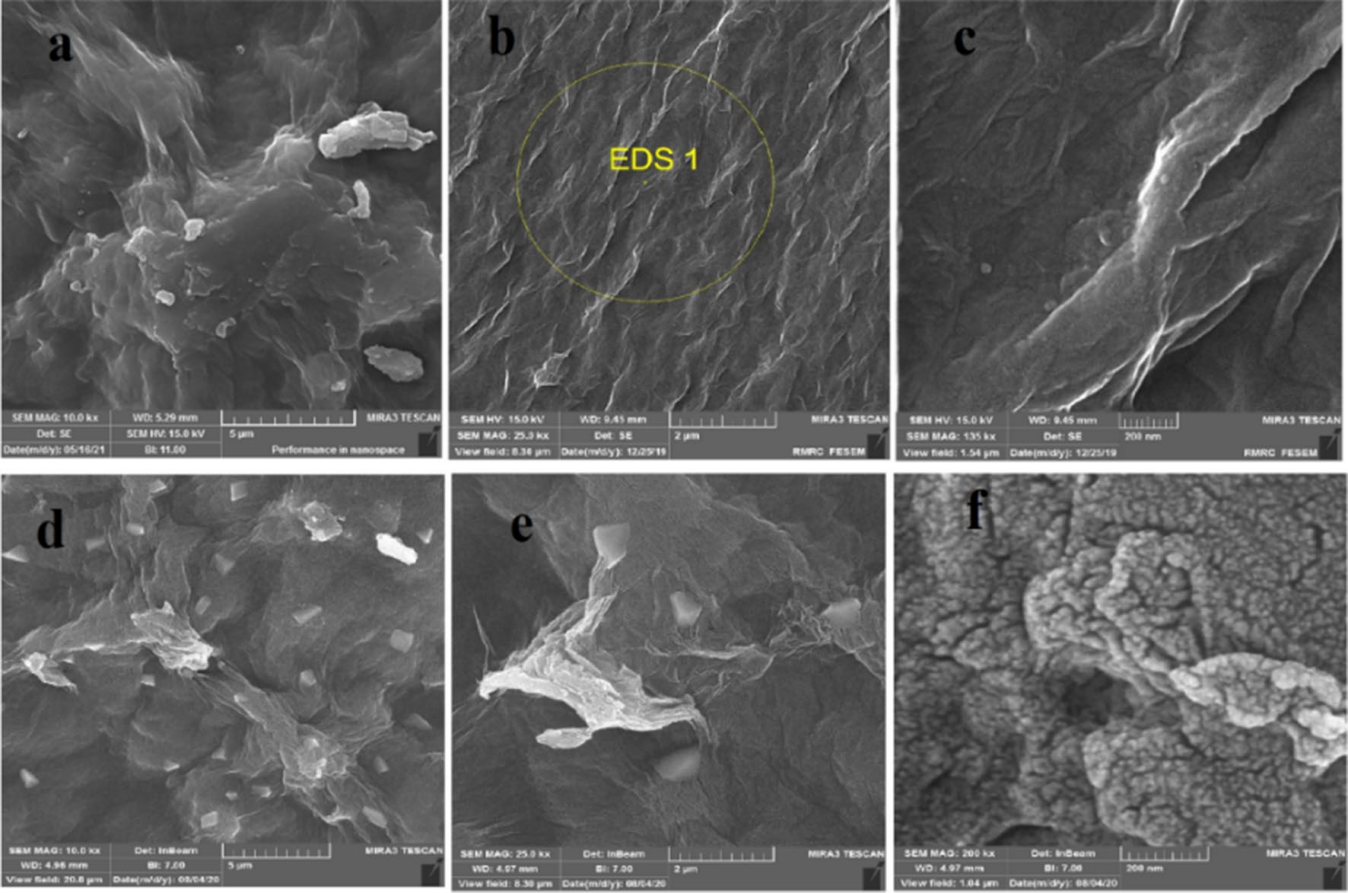
FESEM images of GO (**a**–**c**) and GO-Trp (**d**–**f**).

The surface of GO had wrinkles and irregularities due to the oxygen and hydroxyl groups located on the surface (Fig. 2a–c). By placing of tryptophan on the surface of GO, significant changes in the morphology of the catalyst could be observed. Changes in the GO-Trp surface indicate the modification of GO with the amino acid (Fig. 2d–f). By placing of amino acid to the surface of GO, a three-dimensional porous composite with a high surface area was created. This spongy composite may help to better interaction between the starting materials of the reaction.

#### XRD analysis

**Figure 3:**
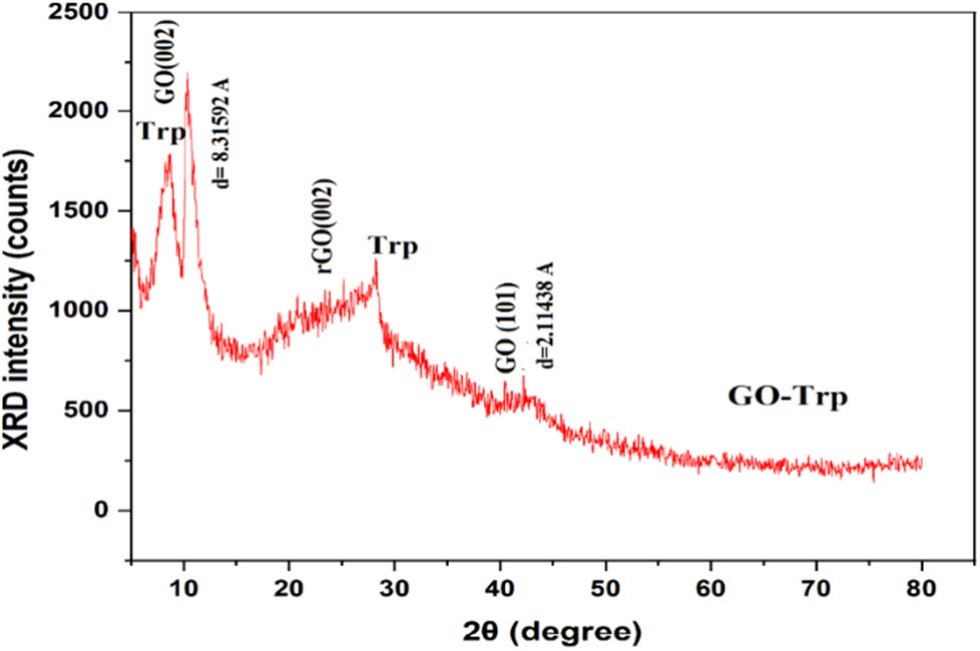
XRD patterns of GO-Trp.

In the XRD pattern of GO, two peaks at 10.35° and 42.37° were observed^46^. Adding of tryptophan to the GO presented three phases in the XRD pattern including GO, rGO, and tryptophan. Peaks positions for GO are at 10.49° and 42.43°, rGO showed a broad peak at 25.07° and tryptophan has two peaks in 8.61° and 28.19°^47–53^. These results indicated that tryptophan has succeeded to some extent reduced the GO. It means the tryptophan has been able to remove some of the hydroxyl groups (–OH) of the GO. For more visibility, the quantitative phase calculation was performed on the GO-Trp sample according to the related equation from the literature (considering the dominant peak for each phase)^54^. The obtained results show ~ 37.83%, 31.49%, and 30.67% for GO, rGO, and tryptophan, respectively. Moreover, the peak position for GO shifted to a higher position after linked to the tryptophan (Fig. 3). According to the Bragg law (nλ = 2dsinθ, λ is X-ray wavelength, d is inter-planner spacing, and θ is the position of diffraction peak), this shift can be a sign of the lower inter-planner d-spacing for GO-Trp in comparison to the GO sample^54^. This decrease can be confirmed with d-spacing values from the XRD patterns (Fig. 3). In addition, GO peaks in the GO-Trp sample show lower intensity compared to the GO sample. This can be indicated lower crystallinity, higher disorders, and more defects by the addition of tryptophan to the GO.

#### XPS analysis

**Figure 4:**
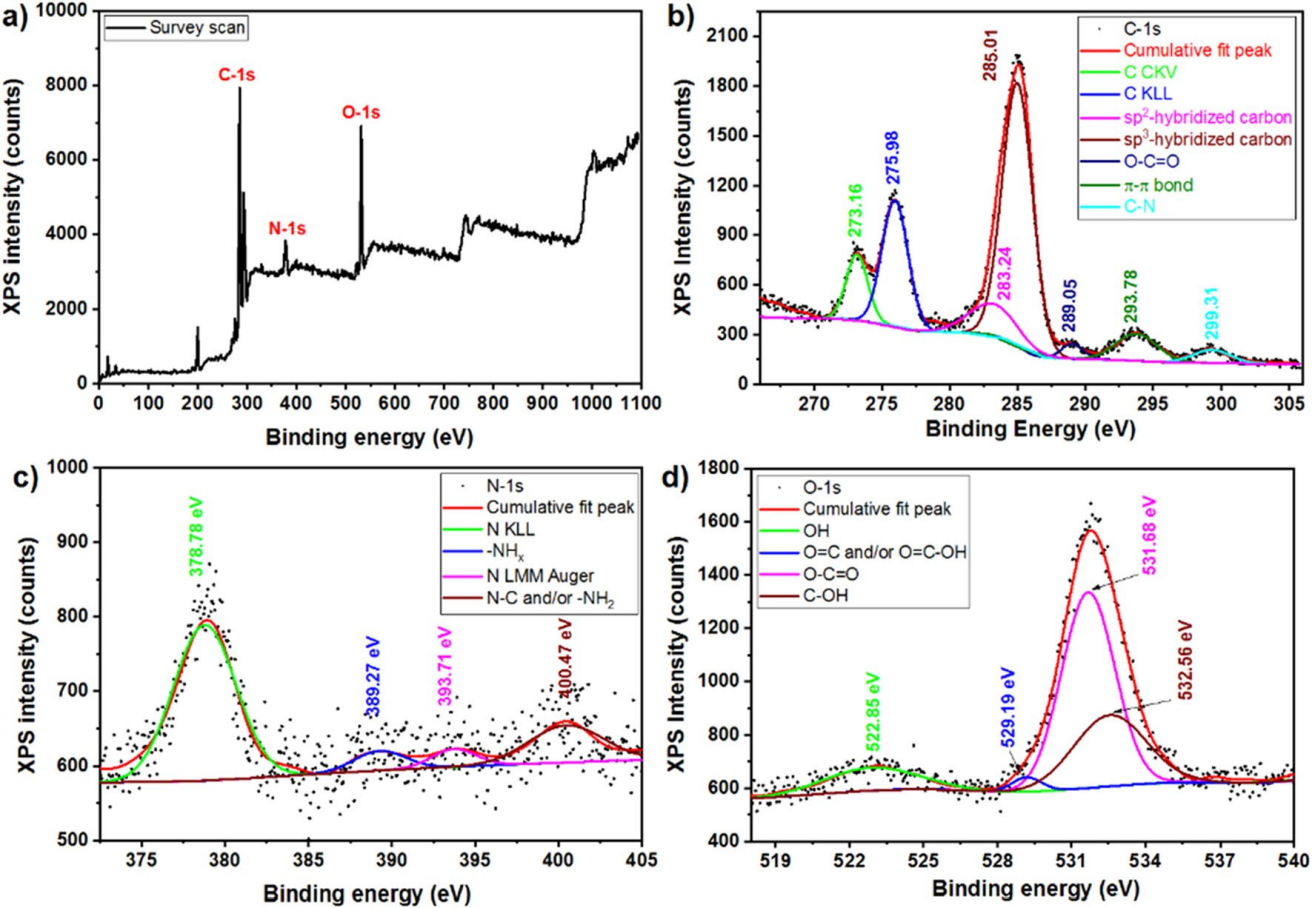
XPS spectra of GO-Trp, survey scan (**a**), partial scan of C-1*s* (**b**), N-1*s* (**c**), and O-1*s* (**d**).

Figure 4 displays the XPS spectra of the GO-Trp sample. The survey scan (Fig. 4a) clearly shows the existence of the C-1*s*, N-1*s*, and O-1*s* peaks. Figure 4b–d presented the partial scans of the mentioned binding energy for more study. These binding energies were de-convoluted using the Gaussian function and the corresponding binding energy and their origination were labelled according to the literature^8,46,55–57^. The different peaks in the C-1*s* in Fig. 4b confirmed the existence of the chemical bonds related to the graphene components (GO and rGO). The obtained chemical bonds in the N-1*s* in Fig. 4c also confirmed the existence of the tryptophan in the catalyst. The study of the partial scan of the O-1*s* (Fig. 4d) beside the previous results indicated the graphene linked to tryptophan from the nitrogen side.

#### EDX analysis

**Figure 5:**
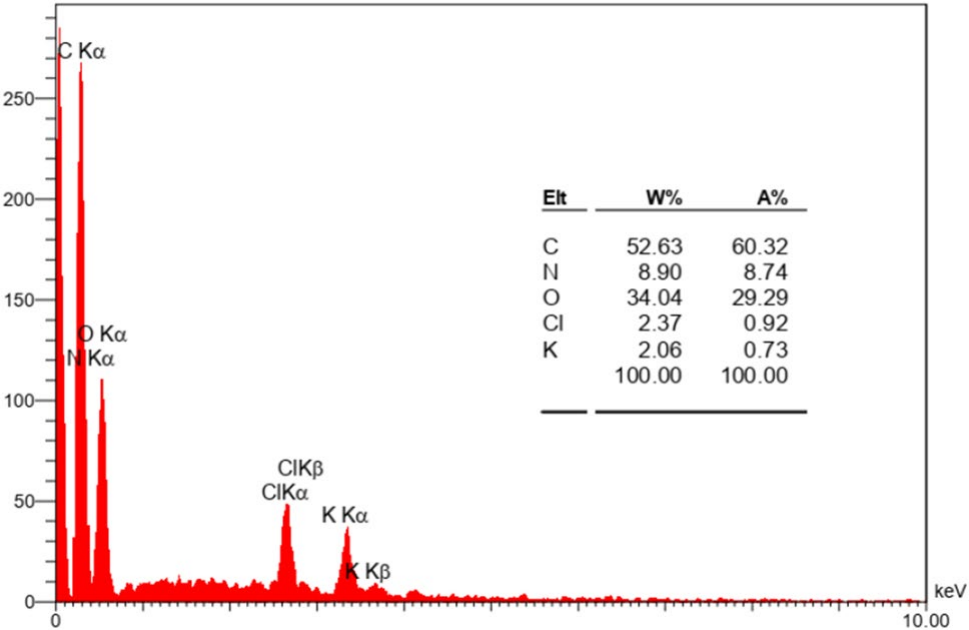
EDX analysis of GO-Trp.

**Figure 6:**
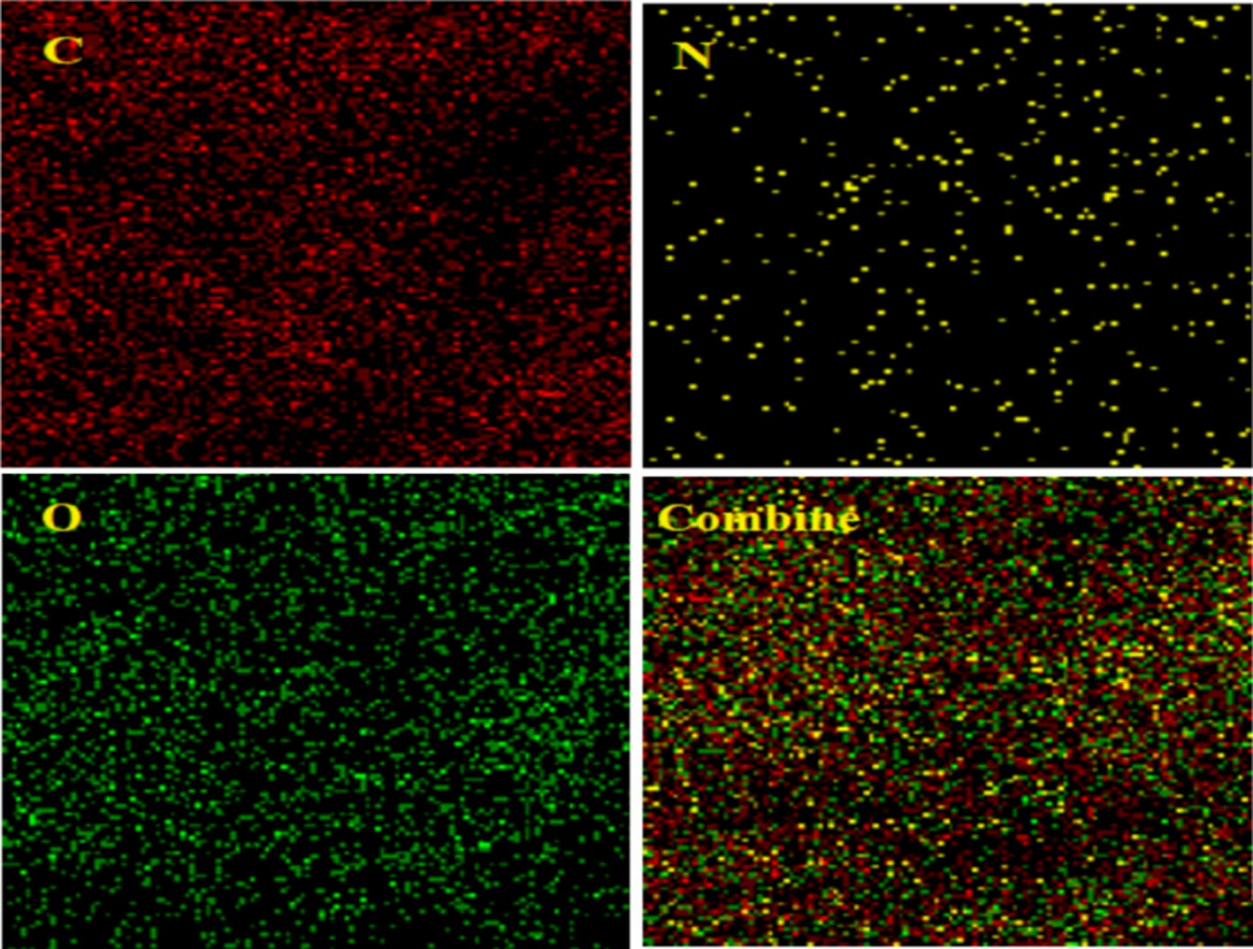
EDX-mapping analysis of GO-Trp.

In this analysis, the initial values of C, N, and O by weight percentage (%W) were shown as 50.37, 13.59, and 35.69, respectively (Fig. 5). According to this analysis, the presence of N, O, and C elements in the catalyst, confirmed the presence of the amino acid in its structure. To examine how the elements in the composite were distributed, a mapping analysis was also performed (Fig. 6). EDX-mapping also showed the presence of N in the composite, indicating the entry of tryptophan into the composite structure. These images also showed the distribution of the elements in the GO-Trp catalyst. The EDX-mapping analysis also shows a good and uniform distribution of N, C, and O elements on the surface of GO.

#### Raman study

**Figure 7:**
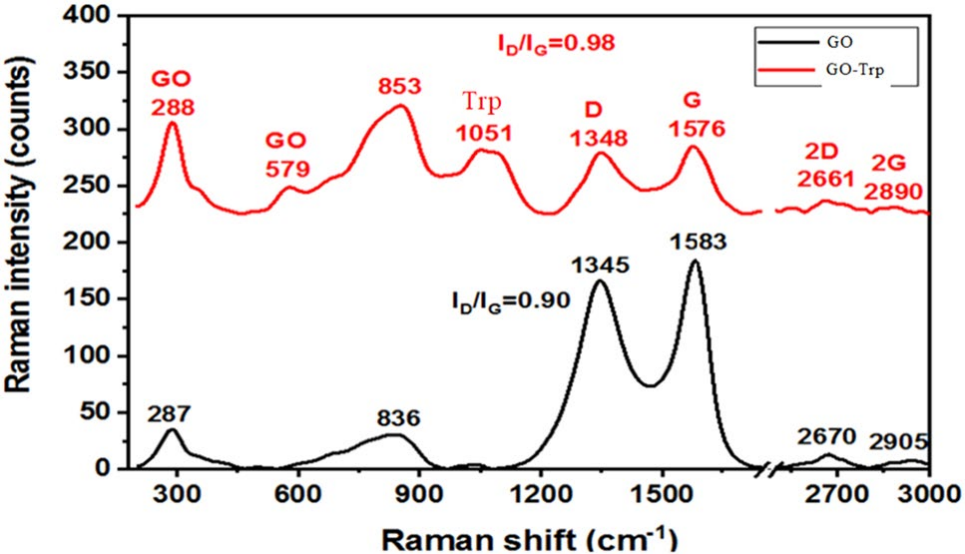
Raman spectra of GO and GO-Trp.

Raman study is a fast and non-destructive method for investigation of the phase purity and structural features of carbon-based materials. Figure 7 represented the Raman spectra of GO and GO-Trp samples. The Raman spectra of the GO sample clearly demonstrated the carbon vibration bands belong to the GO materials^58^. In the GO-Trp Raman spectra, the vibration band of the tryptophan was also observed (~ 1051 cm^−1^)^59^. Moreover, the I_D_/I_G_ is a proper tool for the evolution of the existence of the rGO component in the GO-Trp sample. This ratio for all samples was calculated and shown in Fig. 7. According to the literature^46^, when this ratio was near unity, the reduction of the GO happened. So, the Raman results also confirmed the rGO component formation on the GO-Trp sample in a harmony with the XRD results. In addition, increasing the I_D_/I_G_ ratio can be implied on more defects and disorders in agreement with the XRD results^8,60^.

#### TGA analysis

**Figure 8:**
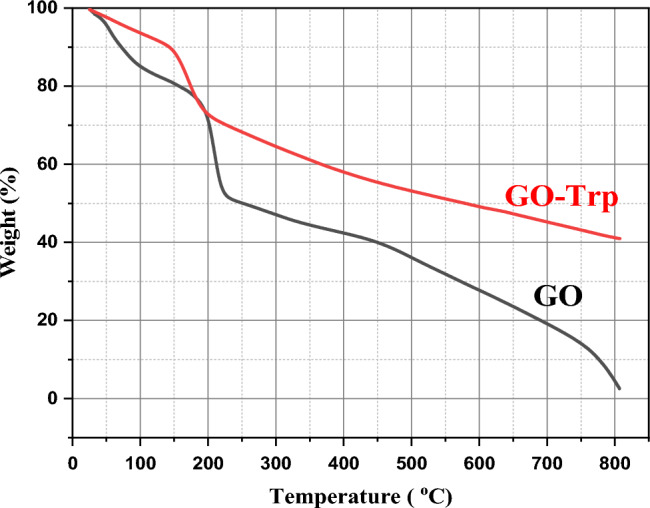
TGA curve of GO and GO-Trp.

TGA analysis can be used to determine the amount of bonded amino acids in this catalyst. At 150–600 °C, covalent bonds between amino acids and GO plates were eliminated. According to the TGA analysis of GO and GO-Trp, amino acid-functionalized GO showed desirable thermal stability (Fig. 8). In TGA analysis of GO, there are a low percent (15%) weight losses at 100 °C, which are mostly caused by moisture evaporation from the graphene oxide. Moreover, the heat treatment’s elimination of GO’s oxygen-containing functional groups is responsible for the main weight loss that happens in the vicinity of 200 °C. As the temperature rises, the GO experiences a thermal reduction and graphene is agglomerated into the graphite form.

In TGA analysis of GO-Trp, at 100–150 °C, the first failure occurred which is related to the removal of water. The second failure occurred at 150 °C, which is related to the removal of oxygen-containing groups. This weight loss led to the production of CO_2_, CO, and water vapour^30^. The residual mass after failure was 40%. Compared to GO, amino acid-functionalized GO had less weight loss due to the reduction in the amount of oxygen-containing functional groups on the surface of graphene flakes. This weight loss was about 18% and is due to the thermal decomposition of organic groups on the GO plate (residual weight 72%). This is evidence of the successful presence of tryptophan molecules on GO plates, which due to the chemical bonds created by tryptophan increased pyrolysis resistance in comparison to GO.

### Optimization of the reaction conditions

To achieve the most favourable conditions for the designed catalyst, three-component reaction of malononitrile, benzaldehyde, and daimedone for synthesis of ***4a*** was selected as a model reaction. Different parameters such as catalyst, solvent, temperature and amount of catalyst were evaluated. In the first step, the model reaction was tested in the presence of GO and GO-Trp in ethanol at 60 °C to confirm the catalytic properties of tryptophan. A comparison of the reaction times and yields showed the effectiveness of tryptophan in the reaction (Table 1, rows 1–2). In the next step, the reaction was performed in different solvents and also solvent-free conditions. According to the results, the best media for the reaction was water/ethanol (1:1) (Table 1, rows 2–6). To determine the best temperature for the reaction, the model reaction was performed at room temperatures and 60 °C. As increasing the temperature had no effect on the reaction progress, the room temperature was selected due to its cost-effectiveness (Table 1, rows 6–7). Finally, to find the required amount of catalyst, the reaction was performed in four different amounts of the catalyst. In 0.03 g of catalyst, the best efficiency and the shortest time were obtained (Table 1, rows 7–10). In conclusion, the best conditions for the synthesis of the desired compounds were selected by performing the reactions in ethanol/water at room temperature with 0.03 g of the catalyst.

**Table 1:** Optimization of the reaction conditions for synthesis of ***4a***.

Entry	Catalyst	Weight of catalyst (g)	Temp. (°C)	Solvent	Time (min)	Yield (%)
1	GO	0.04	60	EtOH	40	55
2	GO-Trp	0.04	60	EtOH	25	96
3	GO-Trp	0.04	60	Solvent-Free	20	92
4	GO-Trp	0.04	60	CH_3_CN	65	41
5	GO-Trp	0.04	60	H_2_O	70	80
6	GO-Trp	0.04	60	EtOH/ H_2_O(1:1)	25	96
7	GO-Trp	0.04	r.t	EtOH/ H_2_O(1:1)	25	96
8	**GO-Trp**	**0.03**	**r.t**	**EtOH/H**_**2**_**O**(1:1)	**25**	**96**
9	GO-Trp	0.02	r.t	EtOH/ H_2_O(1:1)	40	82
10	GO-Trp	0.01	r.t	EtOH/ H_2_O (1:1)	60	70

Significant values are in bold.

### Catalytic activity of GO-Trp in the synthesis of pyran derivatives

**Figure 9:**
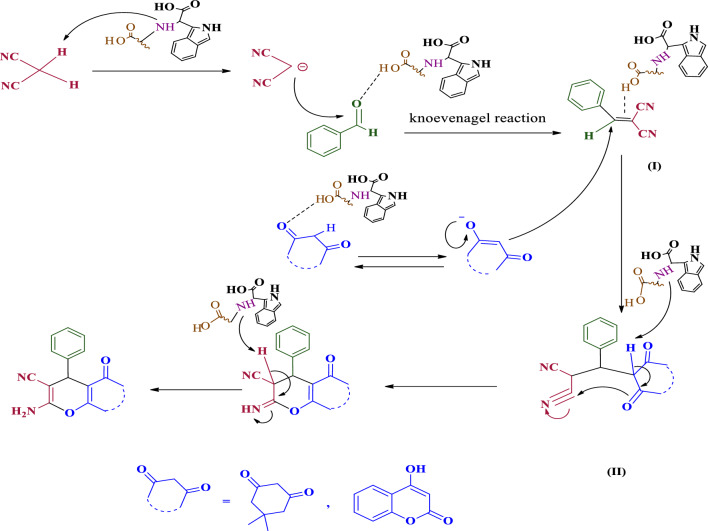
Schematic presentation of proposed mechanism for the synthesis of pyran derivatives in the presence of GO-Trp.

After finding the best conditions some benzo[*b*]pyran (***4a–4g***) and pyrano[3,2-*c*] chromene (***4h–4l***) derivatives were synthesized under the optimized conditions (Table 2). Final products were identified by TLC and comparing their m.p. with those reported in literature. Additionally chemical structures of some compounds were checked by FT-IR, HNMR and CNMR. Their spectra are added to the supplementary file. Our results showed that the presence of electron acceptor groups on the aldehyde increases the efficiency of the reaction compared to the electron donor groups. This is due to increase the electrophilic properties of aldehyde’s carbonyl groups. So, it can interact more easily with the acidic part of the catalyst and provides the conditions for a suitable reaction with nucleophilic groups. The proposed mechanism for the synthesis of the pyran derivatives is shown in Fig. 9. The synthesized nanocomposite with a layered structure and both acidic and basic moieties could progress the reaction easily. At first, the amine part of the catalyst takes a hydrogen from the malononitrile and makes it capable to attack to the carbonyl of aldehyde. In the other hand the oxygen atom of the carbonyl group interacts with the acidic part of the composite. These events causes the aldehyde carbon to become more positive. Next, the dimedone in the presence of the catalyst is converted into the enolate form. This enolate form reacted to the intermediate (I) and produce intermediate (II). Next, the removal of acidic hydrogen by the basic part of the catalyst and an intramolecular reaction lead to the production of the final compound.

**Table 2:** Synthesis of various pyran derivatives under the optimized conditions.

Entry	Products	Time (min)	Yield (%)	M.P. (°C) (Obs.)/(Lit.) [Ref]
***4a***		25	96	230–231/231–233^61^
***4b***		45	87	245–246/246–247^62^
***4c***		15	96	231–232/234–236^62^
***4d***		18	97	235–239/233–235^63^
***4e***		43	85	200–203/203–205^64^
***4f***		30	87	207–210/210–212^61^
***4g***		18	96	215–217/209–211^21^
***4h***		24	93	260–262/260–261^21^
***4i***		32	89	245–250/261–263^21^
***4j***		20	91	271–273/269–270^65^
***4k***		20	97	250–252/255–257^65^
***4l***		20	91	214–218/214–216^65^
***4n***		27	87	250–252/244–246^17^

### Reusability of the catalyst

**Figure 10:**
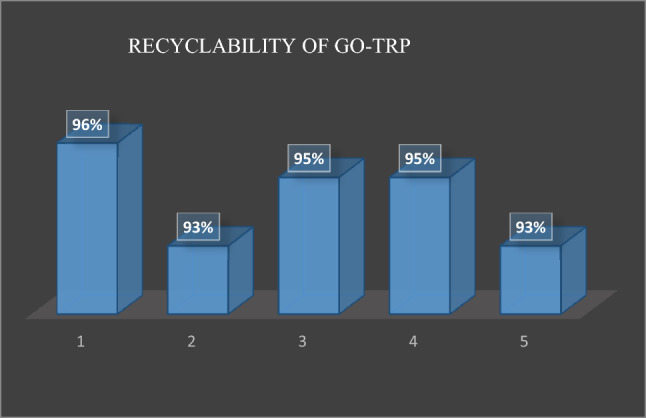
Recyclability of GO-Trp in synthesis of ***4a***.

In order to check the reusability of the catalyst, GO-Trp was isolated from the reaction medium, washed with hot ethanol and then dried at 60 °C overnight. The dried catalyst was used in the subsequent iterations of the model reaction. The results showed that the catalyst efficiency was acceptable until 4 re-uses of the catalyst, which could be due to the complete separation of the reactant residue on the catalyst surface (Fig. 10). The covalent binding of tryptophan to the GO is a key factor in the proper reusability of the catalyst.

**Figure 11:**
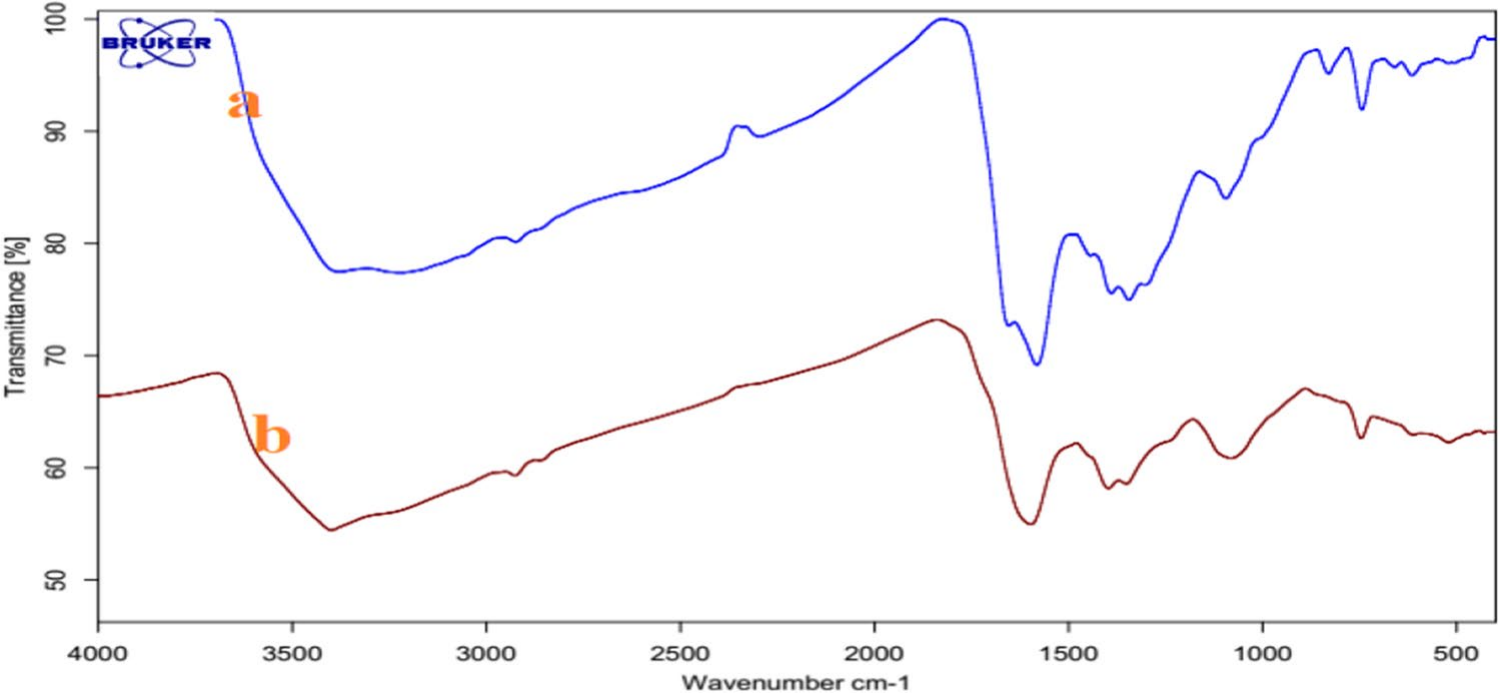
FTIR spectra of fresh GO-Trp (**a**) and used GO-Trp (**b**).

**Figure 12:**
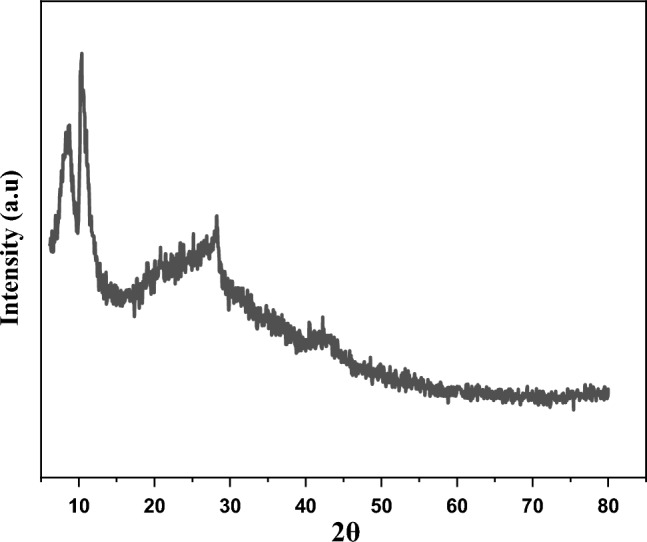
XRD pattern of used GO-Trp.

FT-IR spectra (Fig. 11) and XRD analysis (Fig. 12) from the catalyst before and after the reaction were compared. As can be seen, there was no discernible differences between fresh and used catalyst, proposing the stability of the catalyst.

TON and TOF of the catalyst are equal to 518 mol and 20.7 min^−1^, respectively. The high value of these two parameters indicate desirable efficiency of the catalyst.

### Hot filtration

In continuance of our work, hot filtration test was done on the model reaction to show the recoverability and the heterogeneity nature of GO-Trp. After 5 min from the reaction time, the catalyst was discrete by filtration from the reaction mixture and the reaction was monitored. However, no improvement in the reaction was observed in the filtrate.

### Comparison of the efficiency of GO-Trp with the other reported catalysts

The efficiency of our new catalyst in the model reaction was compared with the other previously reported methods (Table 3). Easy separation and greenness of the catalyst, high yield, short reaction time and performing the reaction in room temperate are advantages of this work compared to the other previous works.

**Table 3:** Comparison of the efficiency of GO-Trp with other catalysts reported for synthesis of pyran derivatives.

Product	Catalyst/Temp/Time (min)/Solvent	Yield (%)
	PC/AgNPs/reflux/35/H_2_O: EtOH (1:1)	82^66^
	BaFe_12_O_19_@ IM/reflux/15/EtOH	96^67^
	MnFe_2_O_4_@SiO_2_NHPhNH_2_–PTA./80 °C/30/Solvent-free	95^64^
	GO-Arg/reflux /10/H_2_O: EtOH (1:1)	93^45^
	Piperazine-GO/50 °C/15/H_2_O–EtOH	95^68^
	Biocompatible Core/Shell Fe_3_O_4_@GA@Isinglass/reflux/15/EtOH	92^69^
	Chitosan‑EDTA‑Cellulose/ r.t./10/EtOH	96^70^
	GO-Trp/r.t. /25/H_2_O: EtOH (1:1)	96 (This work)
	*o*-Benzenedisulfonimide (OBS)/120 °C/50/Solvent-free	85^64^
	PC/AgNPs/reflux/30/H_2_O: EtOH (1:1)	80^66^
	PS-PTSA/80 °C/120/EtOH	94^71^
	GO-Arg/reflux/14/H_2_O: EtOH (1:1)	95^45^
	[Amb]L-prolinate/reflux/20/EtOH	90^72^
	Zr@IL-Fe_3_O_4_ MNPs/100 °C/10/Solvent-free	96^73^
	GO-Trp/r.t./24/H_2_O:EtOH (1:1)	93 (This work)

## Discussions

In summary, the synthesis and identification of a new and naturally catalyst was introduced as an effective and environmentally friendly catalyst to perform a stepwise reaction of pyran derivatives in ethanol–water with high efficiency. GO-Trp was synthesized easily and in a consistent manner with green chemistry in two steps. GO was first synthesized and the amino acid was then located by covalent bonding on GO plates in water at room temperature. The lack of toxic activators such as thionyl chloride, green conditions for temperate, time and solvent were the advantages of this method. Acid-base properties of the catalyst promote the progress of the desired reaction under both basic and acidic conditions. Stabilization of tryptophan molecules on the surface of graphene by covalent bonds, caused porosity, spacing of GO plates, and the formation of a GO layered structure, which improved the catalyst efficiency. The expected structure of the catalyst was verified by IR, XPS, XRD, and Raman analysis. We concluded, the catalysts consisting of both amino and acid moieties can assistance the chemical synthesis. Doing the reaction in green conditions, high efficiency and suitable purity of the products, short reaction time, cheap and non-toxic materials and reusability of the catalyst are other notable advantages of this method. Probably other amino acids can also be used instead of tryptophan to develop some more novel and green catalysts.

## Methods

All chemicals were purchased from Fluka Chemical Co., Switzerland, and Merck Chemical Co., Germany, and were used without further purification. FT-IR spectra were recorded as KBr pills in the 400–4000 cm^−1^ region using Shimadzu FT-IR 8300 spectrophotometer. The characterization of the products was recorded on a Bruker AC 500 Avance DPX spectrometer at 500 MHz for ^1^H NMR and 125 MHz for ^13^C in the presence of TMS. The progress of the reaction was examined using thin-layer chromatography (TLC) on silica gel PolyGram SILG/UV254 plates. Melting points were measured in open capillary tubes in a Buchi melting point B-540 B. The morphology of the samples was studied through FE-SEM, MIRA3TESCAN-XMU. The phase purity of the samples was checked using the XRD analysis using Ni-FILTERED whit Cu-Ka radiation (λ = 1.5406 Å) in the 2θ range from 5° to 80°. XPS spectroscopy was performed by monochromatic Al KR (1486.6 eV) irradiation (a Perkin-Elmer PHI 6000 C/ECSA system). The elemental analysis and distribution were carried out using EDX analysis (recorded by SAMX MIRA II). Raman spectroscopy was accomplished through UniRAM Raman spectrometer with solid-state laser source operating at a wavelength of 785 nm and 200 mW, from 250 to 3000 cm^−1^. Thermogravimetric analysis (TGA) was performed on an STA 504 thermal analysis machine B.

### Synthesis of graphene oxide modified with tryptophan (GO-Trp)

**Figure 13:**
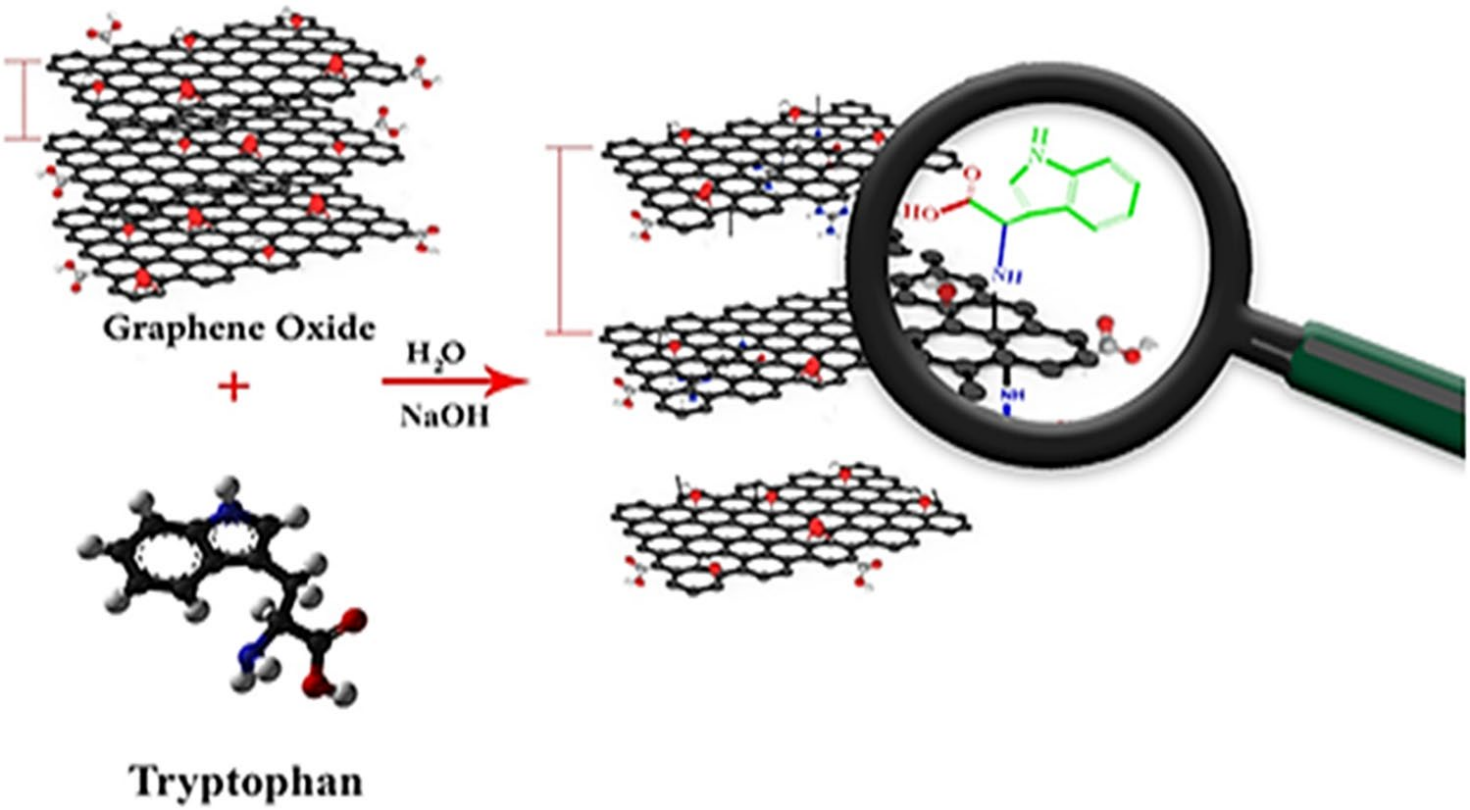
Schematic presentation for synthesis of the GO-Trp.

Initially, GO was prepared according to the Hummer’s method^74^. Then 0.5 g of GO in 20 mL of deionized water was dispersed for half an hour. Then 0.5 g of sodium hydrate followed by 0.5 g of amino acid were added to the stirring reaction vessel. The reaction vessel was allowed to be stirred at room temperature for 24 h. Finally, the catalyst was separated by centrifugation, washed with water/ethanol and dried at 60 °C in a furnace overnight (Fig. 13).

### General procedure for synthesis of the pyran derivatives

**Figure 14:**
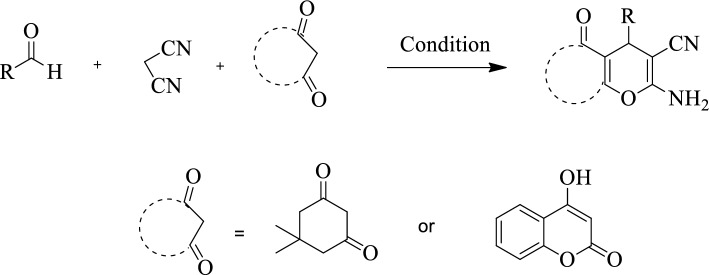
Schematic presentation for synthesis of the pyran derivatives using GO-Trp.

After characterization of the catalyst, its activity for synthesis of benzo[*b*]pyran and pyrano[3,2-*c*] chromene derivatives were investigated. First, malononitrile (0.066 g, 1 mmol), benzaldehyde (1 mmol) and dimedone (0.140 g, 1 mmol) for benzo[*b*]pyran (***4a–4g***) and malononitrile (0.066 g, 1 mmol), benzaldehyde (1 mmol) and 4-hydroxycoumarin (0.162 g, 1 mmol) for pyrano[3,2-*c*] chromene (***4h–4l***) derivatives were poured into a 25 mL flask containing 10 mL water/ethanol. Then, 0.03 g of catalyst was added and the reaction is allowed to take place at room temperature for the required time. The progress of the reaction was checked by TLC. After the reaction completion, the catalyst was separated by centrifugation. The reaction solvent is then evaporated and the resulting precipitate is purified by recrystallization in absolute ethanol. The catalyst washed with hot ethanol and reused in the next reaction (Fig. 14).

### Supplementary Information

Supplementary Figures.

## Data Availability

Data sets generated during and/or analyzed during the current study are available from the corresponding author on request.
