# Supplementary material for: A novel heterogeneous biocatalyst based on graphene oxide for synthesis of pyran derivatives
Source: Sci Rep. 2024 Mar 23;14:6957. doi: 10.1038/s41598-024-57682-y (PMC10960842; doi:10.1038/s41598-024-57682-y)
Supplement: Supplementary file 1 — Supplementary Figures. [file 41598_2024_57682_MOESM1_ESM.docx]

**A novel heterogeneous biocatalyst based on graphene oxide for synthesis of pyran derivatives**

**Leila Amiri-Zirtol^1^, Soghra Khabnadideh^1*^**

*^1^Pharmaceutical Sciences Research Center, Shiraz University of Medical Sciences, Shiraz, Iran*

*Corresponding author: E-mail: [khabns@sums.ac.ir](mailto:khabns@sums.ac.ir)


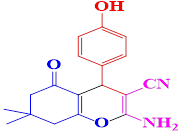

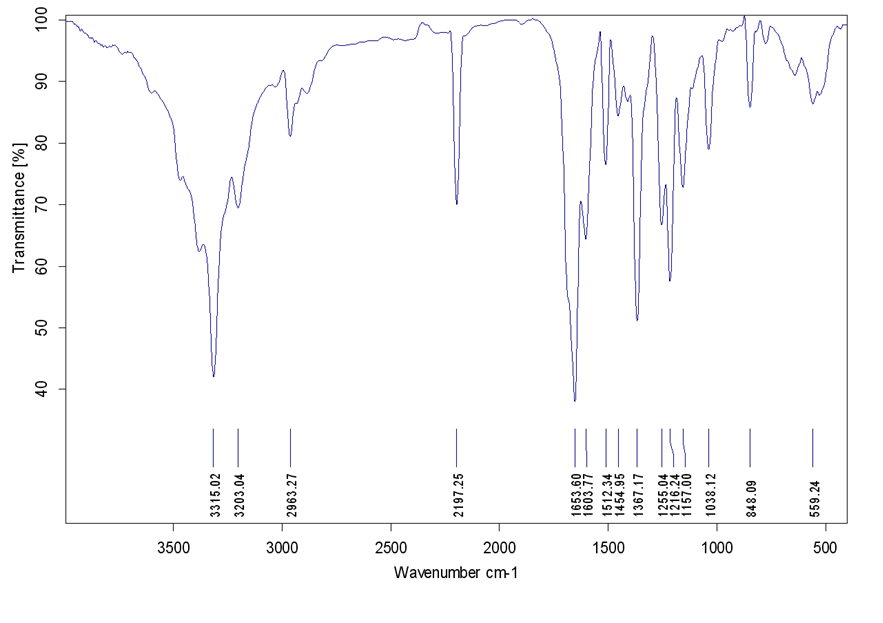


The FT-IR spectrum of 2-amino-4-(4-hydroxy-phenyl)-7,7-dimethyl-5-oxo-5,6,7,8-tetrahydro-*4H*-chromene-3-carbonitrile (4b)


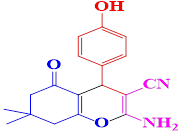

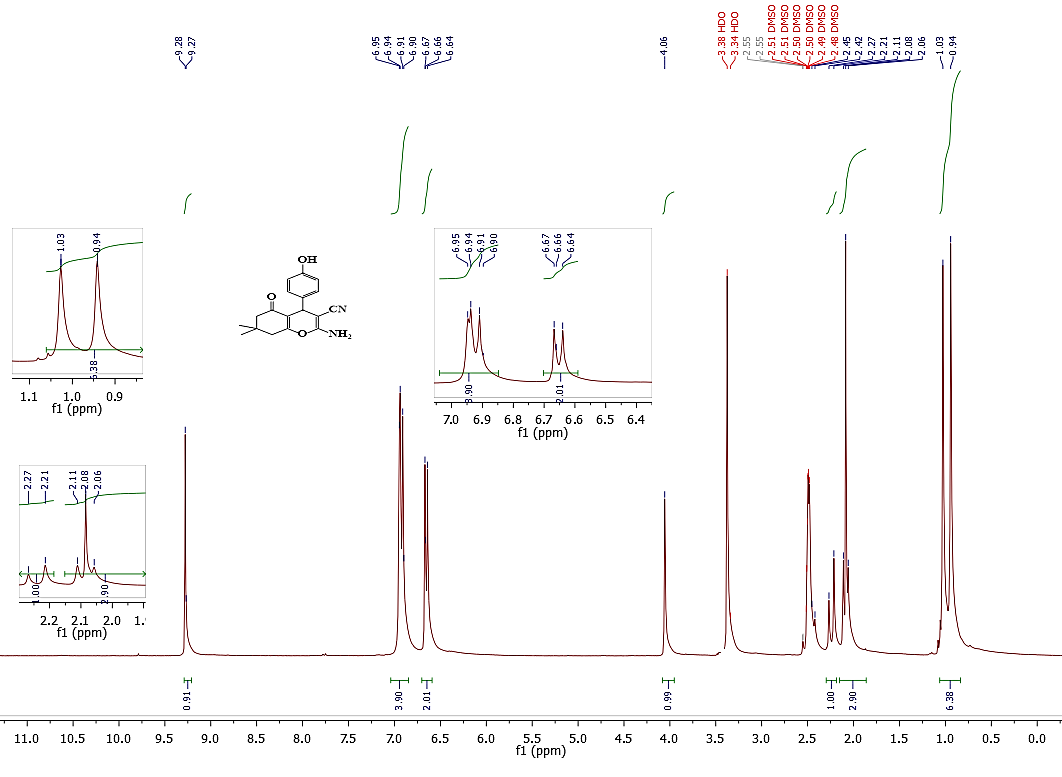


The ^1^H NMR spectrum of 2-amino-4-(4-hydroxy-phenyl)-7,7-dimethyl-5-oxo-5,6,7,8-tetrahydro-*4H*-chromene-3-carbonitrile (4b)


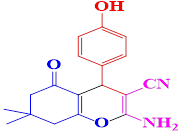

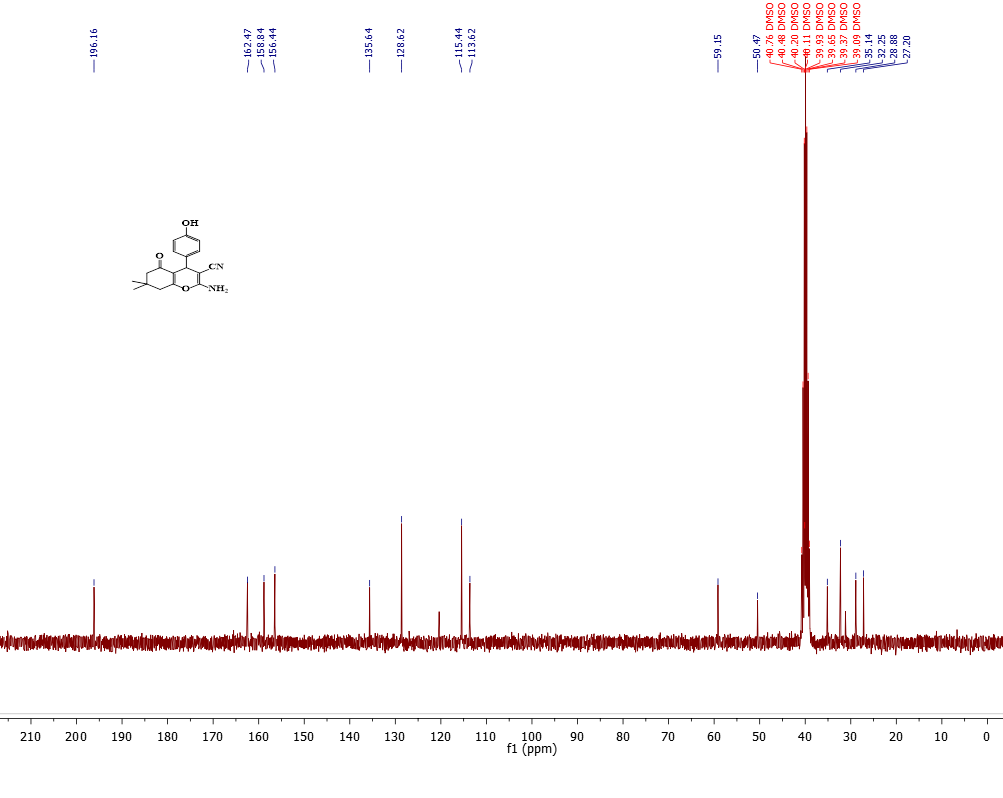


The ^13^C NMR spectrum of 2-amino-4-(4-hydroxy-phenyl)-7,7-dimethyl-5-oxo-5,6,7,8-tetrahydro-*4H*-chromene-3-carbonitrile (4b)


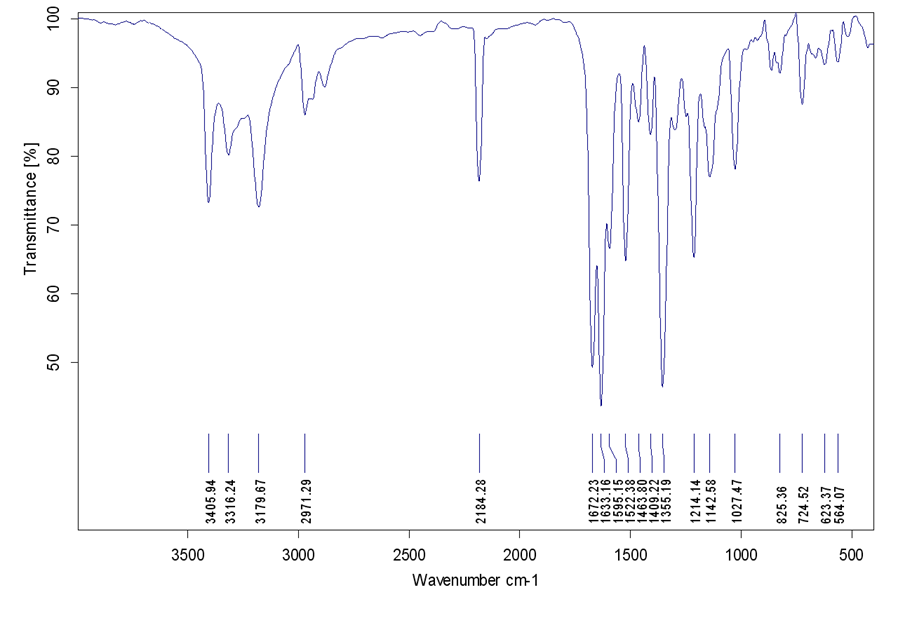

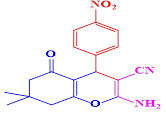


The FT-IR spectrum of 2-amino-7,7-dimethyl-4-(4-nitrophenyl)-5-oxo-5,6,7,8-tetrahydro-*4H*-chromene-3-carbonitrile (4d)


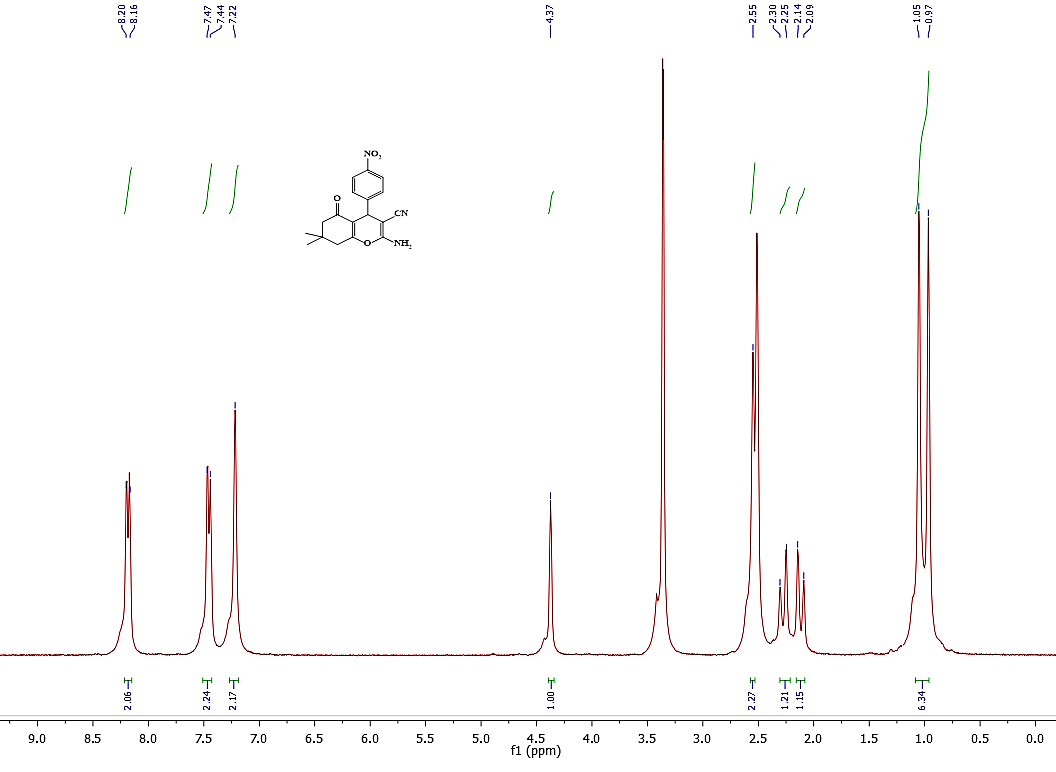


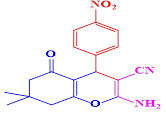


The ^1^H NMR spectrum of 2-amino-7,7-dimethyl-4-(4-nitrophenyl)-5-oxo-5,6,7,8-tetrahydro-*4H*-chromene-3-carbonitrile (4d)


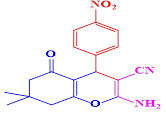

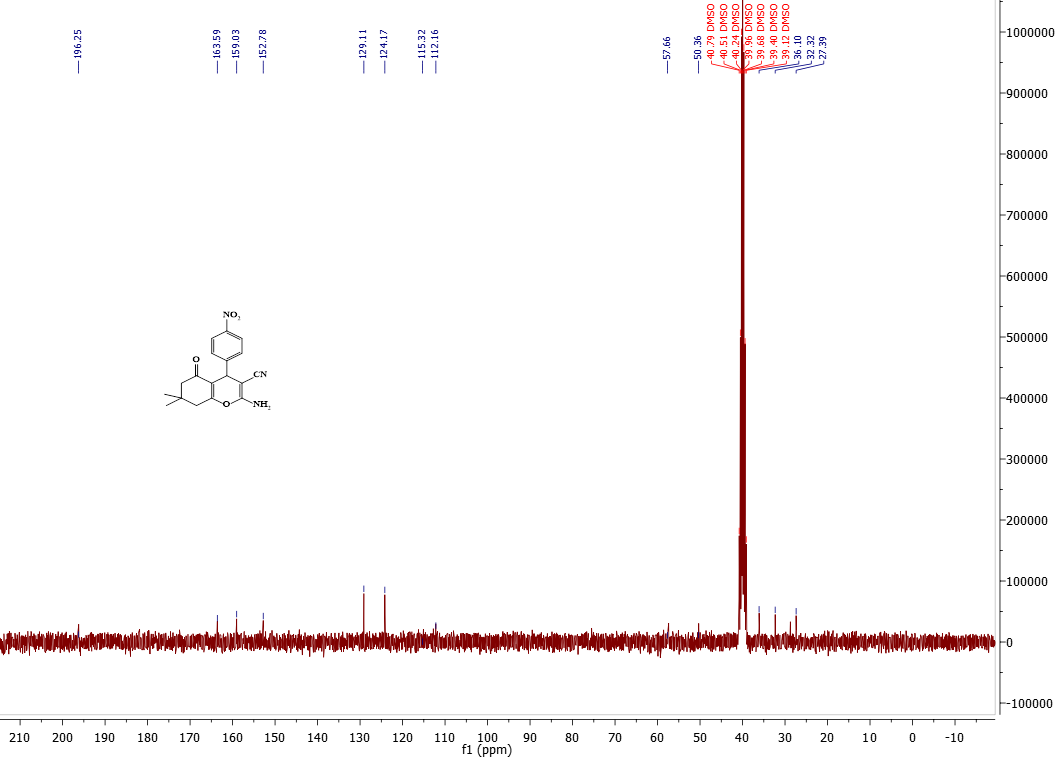


The ^13^C NMR spectrum of 2-amino-7,7-dimethyl-4-(4-nitrophenyl)-5-oxo-5,6,7,8-tetrahydro-*4H*-chromene-3-carbonitrile (4d)


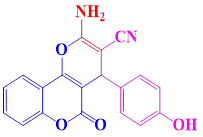

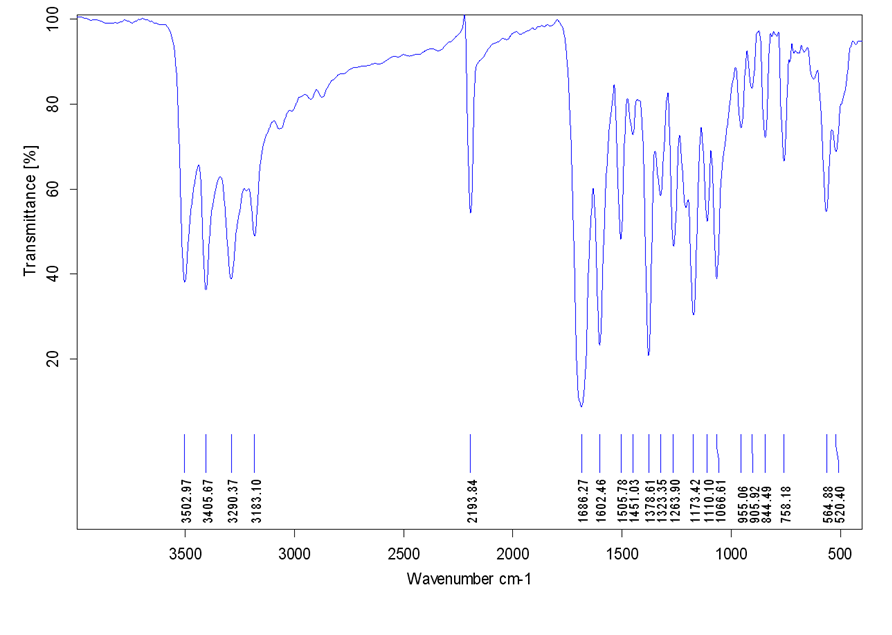


The FT-IR spectrum of 2-Amino-4-(4-hydroxyphenyl)-3-carbonitrile-4,5-dihydro-pyrano[2,3-*c*] chromene-5-one (4i)


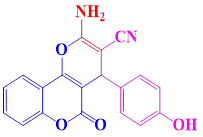

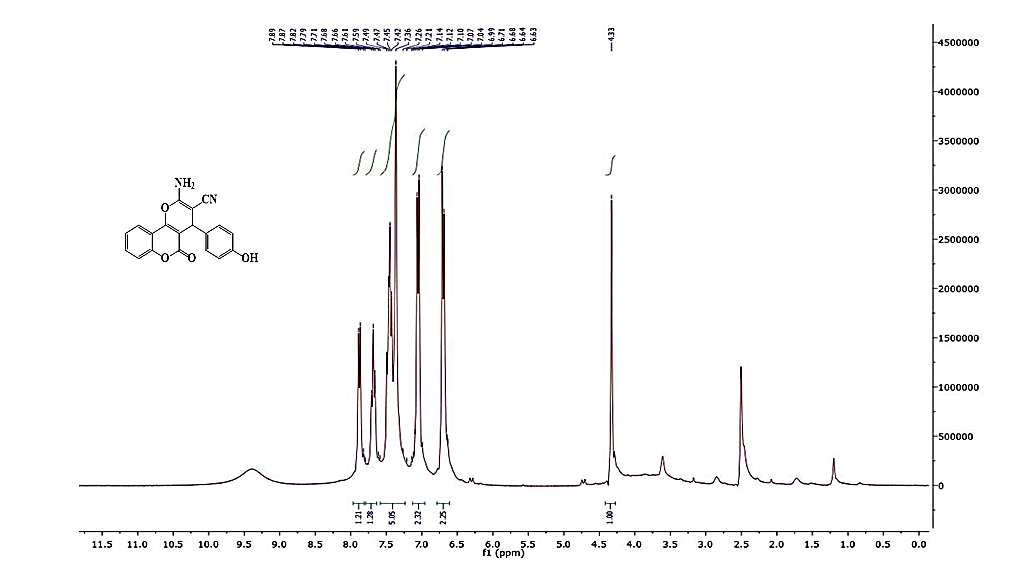


The ^1^H NMR spectrum of 2-Amino-4-(4-hydroxyphenyl)-3-carbonitrile-4,5-dihydro-pyrano[2,3-*c*] chromene-5-one (4i)


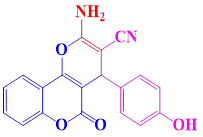

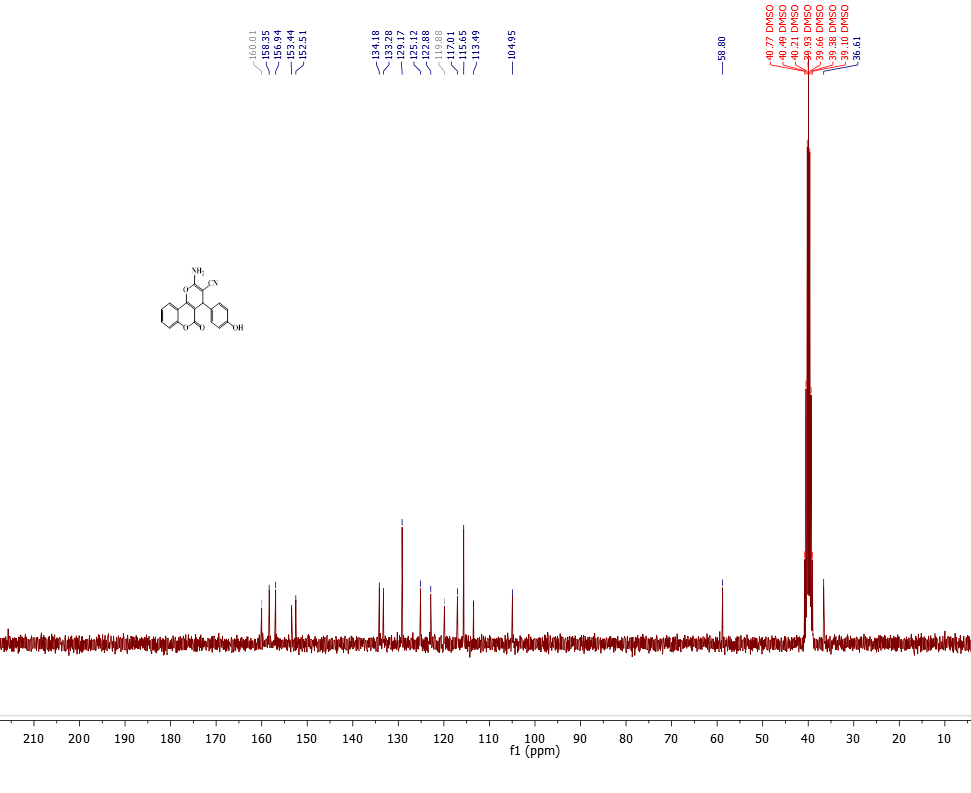


The ^13^C NMR spectrum of 2-Amino-4-(4-hydroxyphenyl)-3-carbonitrile-4,5-dihydro-pyrano[2,3-*c*] chromene-5-one (4i)


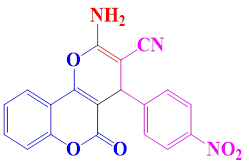

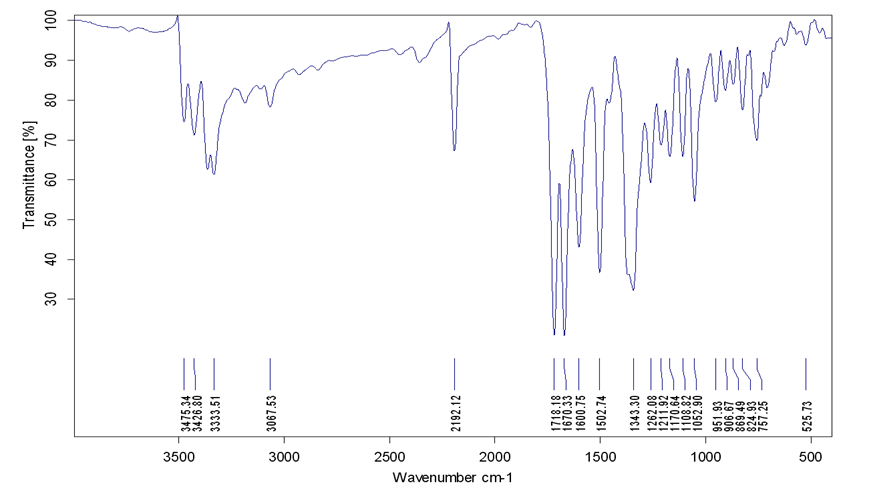


The FT-IR spectrum of 2-Amino-4-(4-nitrophenyl)-3-carbonitrile-4,5-dihydro-pyrano[2,3-*c*] chromene-5-one (4k)


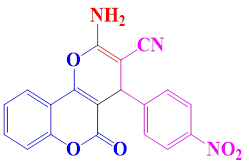

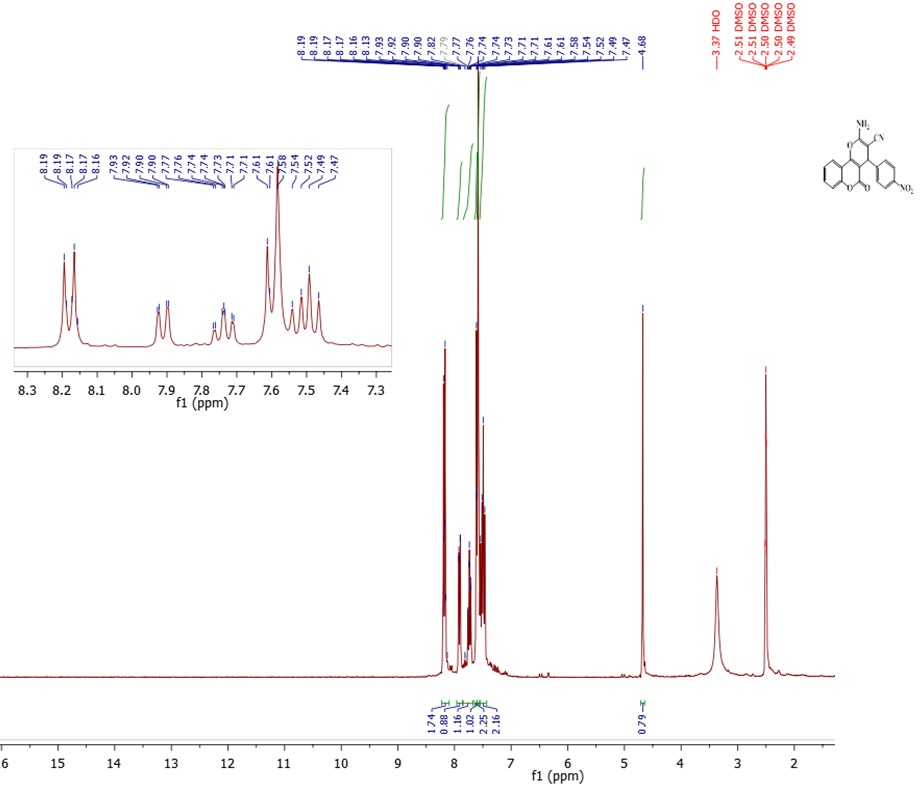


The ^1^H NMR spectrum of 2-Amino-4-(4-nitrophenyl)-3-carbonitrile-4,5-dihydro-pyrano[2,3-*c*] chromene-5-one (4k)


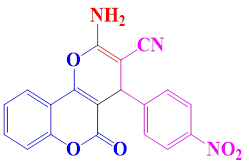

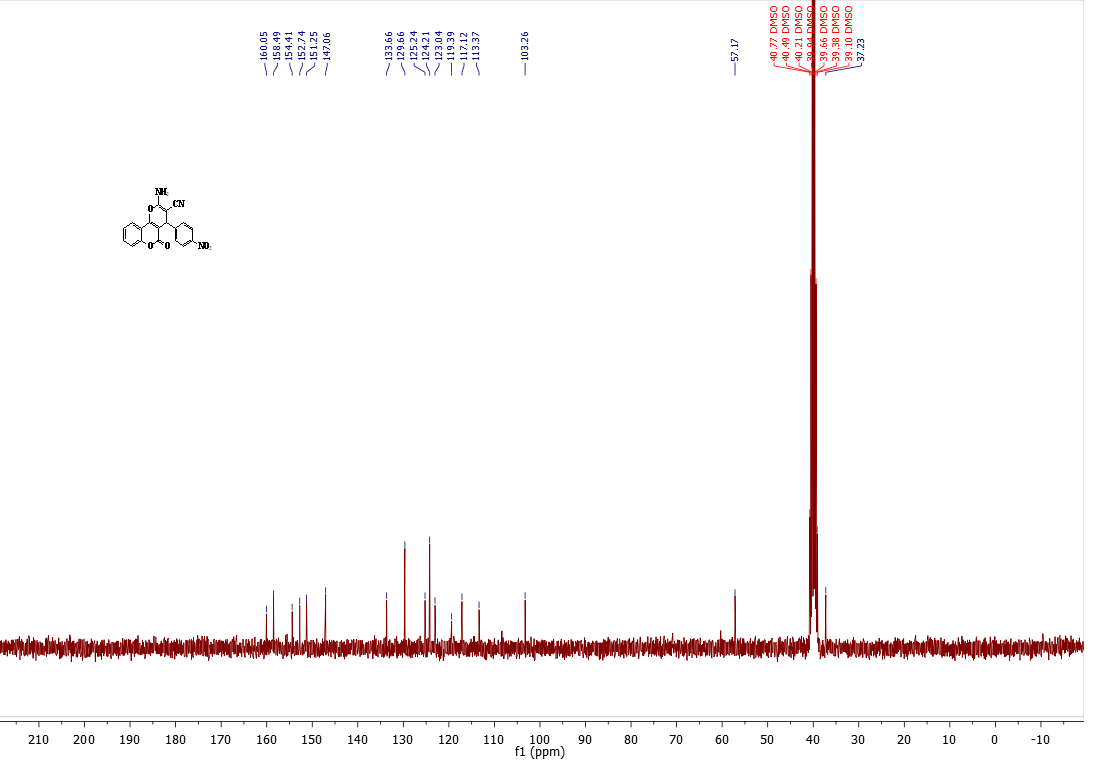


The ^13^C NMR spectrum of 2-Amino-4-(4-nitrophenyl)-3-carbonitrile-4,5-dihydro-pyrano[2,3-*c*] chromene-5-one (4k)
